# Approaches for Reducing the Risk of Early-Life Iron Deficiency-Induced Brain Dysfunction in Children

**DOI:** 10.3390/nu10020227

**Published:** 2018-02-17

**Authors:** Sarah E. Cusick, Michael K. Georgieff, Raghavendra Rao

**Affiliations:** 1 Division of Global Pediatrics, Department of Pediatrics, and Center for Neurobehavioral Development, University of Minnesota, Minneapolis, MN 55455, USA; scusick@umn.edu; 2 Division of Neonatology, Department of Pediatrics, Institute of Child Development, and Center for Neurobehavioral Development, University of Minnesota, Minneapolis, MN 55455, USA; georg001@umn.edu; 3 Center for Neurobehavioral Development, University of Minnesota, 717 Delaware Street SE, Suite 333, Minneapolis, MN 55414, USA

**Keywords:** iron, iron deficiency, iron supplementation, infants, children, neurodevelopment, brain dysfunction

## Abstract

Iron deficiency is the most common micronutrient deficiency in the world. Women of reproductive age and young children are particularly vulnerable. Iron deficiency in late prenatal and early postnatal periods can lead to long-term neurobehavioral deficits, despite iron treatment. This may occur because screening and treatment of iron deficiency in children is currently focused on detection of anemia and not neurodevelopment. Anemia is the end-stage state of iron deficiency. The brain becomes iron deficient before the onset of anemia due to prioritization of the available iron to the red blood cells (RBCs) over other organs. Brain iron deficiency, independent of anemia, is responsible for the adverse neurological effects. Early diagnosis and treatment of impending brain dysfunction in the pre-anemic stage is necessary to prevent neurological deficits. The currently available hematological indices are not sensitive biomarkers of brain iron deficiency and dysfunction. Studies in non-human primate models suggest that serum proteomic and metabolomic analyses may be superior for this purpose. Maternal iron supplementation, delayed clamping or milking of the umbilical cord, and early iron supplementation improve the iron status of at-risk infants. Whether these strategies prevent iron deficiency-induced brain dysfunction has yet to be determined. The potential for oxidant stress, altered gastrointestinal microbiome and other adverse effects associated with iron supplementation cautions against indiscriminate iron supplementation of children in malaria-endemic regions and iron-sufficient populations.

## 1. Introduction

Iron is essential for the normal development and function of all tissues in the body. Iron-containing heme proteins (Hemoglobin [Hgb] and cytochromes) participate in tissue oxygen delivery and energy metabolism. In the brain, iron and iron-containing enzymes are necessary for neuronal and glial energy metabolism, myelin synthesis and neurotransmission [1]. From a public health point of view, iron deficiency is the most common micronutrient deficiency in the world [2]. Women of childbearing age and preschool age children are particularly vulnerable. In addition to being the most common cause of anemia, iron deficiency during the late prenatal and early postnatal periods is a risk factor for long-term neurodevelopmental abnormalities [1,3,4]. Thus, early detection and prompt treatment of iron deficiency is of public health significance. Conversely, excess iron supplementation is associated with growth failure, altered gastrointestinal microbiome and other adverse effects in children, suggesting the need for a balanced approach. In the following sections, we review the causes and adverse neurological consequences of iron deficiency in infants and children, the current screening and treatment recommendations, and recent advances in diagnosis and treatment strategies for preventing iron-deficiency-induced adverse neurological effects.

## 2. Children At-Risk of Iron Deficiency

Children are at risk of iron deficiency at three time points: in the late prenatal and neonatal period; between 6 and 24 months of age; and at adolescence. The former two time periods (first 1000 days; hereafter called early-life iron deficiency) coincide with the period of rapid brain growth and development and can negatively impact neurodevelopment. The common causes of iron deficiency in the fetal and neonatal period are maternal iron-deficiency anemia, preterm birth, and gestational complications such as maternal diabetes mellitus, intrauterine growth restriction, maternal smoking, maternal obesity and inflammation. Consumption of a diet that is low in iron and/or contains iron binders and chronic gastrointestinal blood loss due to cow milk intolerance or hookworm infestation are the common causes of iron deficiency in the post-neonatal period. Iron deficiency during adolescence is common in female athletes due to a combination of insufficient dietary iron intake, menstrual blood losses and exercise-induced hepcidin upregulation [5]. In contrast to early-life iron deficiency, the neurological effects of iron deficiency at adolescence and beyond are reversible with iron supplementation [6], likely because the brain is fully developed by this age.

## 3. Early-Life Iron Deficiency and Brain Dysfunction

### 3.1. Inter-Organ Prioritization of Iron in Early-Life Iron Deficiency

The majority (60–70%) of the total body iron is in the red blood cells (RBCs) as Hgb; the rest is in tissues and storage form [7]. During negative iron balance in early life, available iron is prioritized to the RBCs over all other organs, including the brain [8,9,10,11]. This risk is greatest in the late fetal through infancy periods when the iron needs of growth competes with those of erythropoiesis. Studies in infant humans [8,12], monkeys [13], lambs [9,14] and rats [15] demonstrate that brain iron is reduced prior to the appearance of anemia. Similar prioritization (RBC before brain) also occurs during recovery from iron deficiency. The Hgb is normalized before the brain becomes iron replete in anemic infant rats and monkeys during treatment [13,15]. The slower recovery of brain iron is of concern, because iron transport across the blood–brain barrier is developmentally regulated [16] and delaying iron treatment misses out this window of opportunity, leaving the brain iron deficient [17]. Residual brain iron deficiency is likely responsible for the persistent neurological deficits in children with a history of early-life iron deficiency [3,4]. 

### 3.2. Neurological Sequelae of Early-Life Iron Deficiency

An in-depth review of the neurological effects of early-life iron deficiency is beyond the scope of this review. Excellent reviews are available elsewhere [1,18]. Briefly, late prenatal and neonatal iron deficiency is associated with altered temperament [19], abnormal recognition memory [20,21], and mental and psychomotor deficits in full-term infants [22], and abnormal neurological reflexes [23] and auditory brain-stem response in preterm infants [24]. An association between fetal iron deficiency and schizophrenia has been reported [25]. Postnatally, iron deficiency between 6 and 24 months of age is associated with lower IQ, slower processing speed, deficits in attention, motor, cognitive and behavioral functions, and disrupted sleep–wake rhythm [26]. While early treatment improves motor performance, behavioral deficits often persist into adulthood [27].

### 3.3. Biology of Abnormal Neurodevelopment in Early-Life Iron Deficiency

Rodent and non-human primate models demonstrate that early-life iron deficiency anemia causes impaired cerebral energy metabolism [15,28], hypomyelination, altered monoamine metabolism [29], abnormal synaptic architecture, and suppression of growth factor expression [15,30,31,32,33,34]. The hippocampus, striatum and cerebellum are targeted. Similar effects are seen with non-anemic hippocampus-specific iron deficiency, suggesting that brain tissue iron deficiency is primarily responsible for the adverse effects. The animal studies also highlight the importance of timing iron treatment for reversing the adverse neurological effects. Whereas early iron treatment corrects brain iron deficiency and restores brain metabolism and function [35], late treatment after the onset of anemia fails to produce similar beneficial effects [36], even when higher than the standard iron doses are used [36,37]. Thus, early detection and treatment is important for ensuring the normal neurodevelopment of children at risk of early-life iron deficiency.

## 4. Screening and Treatment Strategies

### 4.1. Current Recommendation

The American Academy of Pediatrics currently endorses universal screening for anemia at 12 months of age through the determination of Hgb and an assessment of risk factors for iron deficiency [38]. If anemia (Hgb < 110 g/L) is present, then additional screening for iron deficiency by measuring serum ferritin and C-reactive protein (CRP) levels (to rule out false elevation in serum ferritin due to inflammation) or reticulocyte Hgb concentration is recommended [38]. This strategy is unlikely to ensure neuroprotection. As mentioned above, anemia is the end-stage state of iron deficiency due to the prioritization of iron to the RBCs over other organs [8,14,39,40]. The brain is already iron deficient by the time anemia is diagnosed. Animal studies show that it is brain-tissue iron deficiency, independent of anemia, that is responsible for the neurological deficits [17,41]. Screening for anemia also fails to detect non-anemic iron deficiency, which is 3-fold more common than iron deficiency anemia even in the United States [42], and a risk factor for neurological impairments [43]. Furthermore, the laboratory tests used for screening (Hgb, ferritin and reticulocyte Hgb) are biomarkers of hematological changes. Our recent study in non-human primates demonstrates that these hematological and iron panel biomarkers are not sensitive for detecting brain iron deficiency and cerebral metabolic dysfunction in the pre-anemic period [44]. Finally, starting iron treatment after the onset of anemia does not correct the adverse neurological effects, even when an extended duration of iron therapy is used [45].

### 4.2. Potential Biomarkers of Brain Dysfunction in Early-Life Iron Deficiency

We have previously reported that a cord blood ferritin < 35 µg/L predicts brain iron deficiency and dysfunction as indexed by impaired recognition memory at birth, and lower psychomotor development at 1 year of age in full-term infants with iron deficiency due to maternal gestational diabetes [20]. A cord blood ferritin concentration ≤75 µg/L correlates with slower auditory brainstem-evoked responses that are suggestive of reduced auditory tract myelination in the newborn period [24,46]. A cord blood zinc protoporphyrin/heme (ZnPP/H) ratio > 118 µM/M predicts worse recognition memory at 2 months [21]. Unfortunately, a similar association between a serum iron panel index and brain iron deficiency and dysfunction beyond the newborn period has yet to be determined. Reticulocyte Hgb content is the strongest predictor of iron deficiency and response to iron supplementation in children [47,48]. Unpublished studies in neonatal rats from our lab suggest that reticulocyte Hgb is a sensitive peripheral biomarker of impending brain iron deficiency. Validation in human infants is necessary before reticulocyte Hgb could be recommended as a screening tool. Our studies in non-human primate models of infantile iron deficiency suggest that proteomic and metabolomic analysis of biofluids (serum and cerebrospinal fluid) may provide sensitive biomarkers of impending brain metabolic dysfunction in the pre-anemic period [44]. It is important to note, however, that while all of these biomarkers appear to be sensitive for detecting early-life iron deficiency-induced brain dysfunction, currently there is no evidence that instituting iron supplementation based on these biomarkers will prevent or reverse the adverse neurological effects.

## 5. Prevention of Early-Life Iron Deficiency-Induced Brain Dysfunction

Given the difficulties with early detection of brain dysfunction and the ineffectiveness of iron treatment started after the onset of anemia in reversing the neurological deficits, strategies aimed at prevention of early-life iron deficiency are of the utmost importance and potentially should begin with ensuring adequate iron accretion by the fetus. Currently, routine iron supplementation via diet (e.g., iron-fortified cereal) or medicinal iron is recommended for full-term breastfed infants from 4 months of age [38]. In children aged 4–24 months, daily iron supplementation improves hematological status [42,49]. Mental and psychomotor performances are not affected. This lack of beneficial effect on neurodevelopment with iron supplementation has been used as an argument for continuing with the current screening recommendation [50]. However, it is also possible that waiting until 4 months of age to begin iron supplementation may have been too late for preventing brain iron deficiency and associated adverse effects in those at risk of early-life iron deficiency (e.g., those born with low iron stores). Consistent with this possibility, previous studies have demonstrated that iron supplementation using an iron-containing formula within a month of birth improves psychomotor development of at-risk infants [51,52]. Since low iron endowment at birth predisposes to iron deficiency in early-infancy [53], measures that enhance iron stores before and/or soon after birth are likely to be beneficial.

### 5.1. Ensuring Early-Life Iron Sufficiency

#### 5.1.1. Maternal Iron Supplementation

Maternal iron supplementation during pregnancy is a cost-effective method of ensuring iron sufficiency in the mother–infant dyad. The Institute of Medicine recommends that women consume 27 mg/day of iron during pregnancy [54]. However, most women in low- and middle-income countries need additional iron to prevent iron deficiency and maintain adequate stores. Typically, 30–60 mg of elemental iron per day is recommended, with up to 120 mg of elemental iron daily for those with anemia [55,56]. A meta-analysis of 44 trials involving more than 40,000 women in 2015 showed that daily oral iron supplementation during pregnancy reduces maternal anemia by 70%, iron-deficiency anemia by 67%, and iron deficiency by 57% at term gestation [56]. Women receiving iron were more likely to have higher Hgb at delivery and in the postpartum period, and less likely to have low birth weight and preterm infants, compared with those not receiving iron supplementation [56]. An additional theoretical benefit of maternal iron supplementation is better mother–infant interaction due to an improved iron status of the mother. However, compliance with iron supplementation may be poor due to a lack of education and side effects associated with enteral supplementation. In a prospective study in north-east India, the incidence of maternal anemia during pregnancy was 90% due to a combination of the consumption of an iron-poor diet, the habit of drinking large quantities of black tea (which binds to iron in the intestinal lumen and prevents its absorption) with meals, and poor compliance with recommended iron supplementation [57]. There is also a potential for adverse effects with excessive iron supplementation during pregnancy. The above-mentioned meta-analysis found that mothers on iron supplementation were more likely to have Hgb concentration of 130 g/L, a value associated with maternal and fetal adverse effects [56]. Thus, there is a need for considering methods beyond maternal iron supplementation for enhancing offspring’s iron stores. Two examples of such a strategy are delayed clamping or milking of the umbilical cord and early iron supplementation.

#### 5.1.2. Delayed Clamping or Milking of the Umbilical Cord

Delaying clamping of the umbilical cord for 30–45 s after birth is an effective method of increasing Hgb concentration and iron stores in healthy full-term infants. Whereas the improvement in Hgb is limited to the first 24–48 h after birth, the beneficial effects on iron stores last at least until 6 months of age [58]. A similar beneficial effect on iron stores is also seen with immediate clamping of a long segment of the umbilical cord followed by milking it towards the infant [59]. This strategy is useful in situations where delayed clamping of the umbilical cord is not feasible (for example, when there is a need for resuscitation of the infant at birth). The beneficial effects on iron stores were present in infants of both anemic mothers and non-anemic mothers [59], suggesting that the procedure could be undertaken universally in areas where maternal gestational iron deficiency is common. An association between delayed cord clamping and improved scores in fine motor and social domains at 4 years of age in boys has been reported [60].

#### 5.1.3. Initiation of Supplementation Earlier than the Recommended Period

Beginning iron supplementation earlier than the recommended 4 months of age may be beneficial in infants at risk of early-life iron deficiency. In full-term breastfed infants, 7–7.5 mg per day of elemental iron from 1 to 6 months of age leads to higher Hgb and improved iron status at 6 months of age, and better visual acuity and psychomotor development at 13 months of age [52,61]. In areas where iron deficiency is prevalent, starting iron supplementation even earlier may be beneficial. Unpublished data suggest that iron supplementation in a dose of 2 mg/kg daily started on the second day after birth and continued until 6 months improves iron stores and motor development at 6 months of age in breastfed full-term infants in regions with high prevalence of iron deficiency (Bora, personal communication). Whether such supplementation leads to better long-term neurodevelopment has yet to be determined.

## 6. Potential Risk with Universal Iron Supplementation in Children

Public policy approaches to address common and potentially dangerous nutrient deficiencies include fortification and universal supplementation. Folate supplementation of grains and iodide supplementation of salt represent two such successful campaigns that have resulted in subsequent reductions in morbidity from neural tube defects and hypothyroidism/goiter. The decision to universally supplement or fortify with a nutrient takes into consideration the risks and benefits of the nutrient. While no apparent harm has occurred through folate and iodide supplementation, the case for universal supplementation of iron is more difficult because of emerging evidence of the potential toxicities of iron. Two populations at risk of this complication are discussed below.

### 6.1. Iron Supplementation of Children in Malaria-Endemic Areas

In many of the same regions where iron deficiency is most prevalent, malaria is also endemic. The potential danger of giving iron to children living in malaria-endemic regions was brought to the world’s attention by a large, randomized, placebo-controlled trial of prophylactic iron supplementation for young children living on malaria-endemic Pemba Island, Tanzania [62]. This trial was the first large-scale study to test the former recommendation of the World Health Organization (WHO) for daily, universal iron supplementation of children living in areas where the prevalence of anemia was 40% or greater. The Pemba study was stopped early due to an observed increased risk of hospitalizations and deaths among children who received iron. The results of a sub study of the larger study, which included more specific iron-status measures such as ZnPP, as well as more immediate access to prompt malaria diagnosis and treatment, demonstrated that children who were anemic or who had an elevated ZnPP (reflective of iron deficiency) had a significantly lower rate of serious adverse events compared with iron-deficient children who did not receive iron. 

With more than 30,000 children enrolled, the Pemba trial was a landmark study, and its results shook the global nutrition world, changing the policy and practice of giving iron to the tens of millions of children around the world who live in malaria-endemic areas. The most recent of three Cochrane reviews on the topic that were conducted after the Pemba study found (as did the other two reviews) no harmful effect of iron in malaria-endemic area when iron is given in conjunction with malaria management services [63], subsequent studies continued to underscore the potentially dangerous interaction of iron with malaria and other infections. One study of iron-containing multiple micronutrient supplementation reported an increased risk of malaria episodes in iron-deficient Tanzanian children [64], and another study of iron-containing micronutrient powder supplementation reported an increased risk of diarrhea and respiratory illness among Pakistani children [65]. A large birth cohort study in a malaria-endemic area of Tanzania in which no iron was given found an increased risk of all-cause mortality among children who remained iron-replete in the first four years of life compared with children who developed iron-deficiency [66]. More than a decade later, the best way to help young children living in malaria-endemic areas to safely maintain a healthy iron status in order to protect brain development thus remains unclear, although significant research has helped elucidate the pathophysiology of the interaction between iron and malaria, clarifying several potential intervention strategies. 

A meta-analysis of more than 55 randomized controlled trials of iron supplementation in children reported that the Hgb response to iron is diminished in malaria-endemic areas [67]. This finding is in line with malaria itself as an important cause of anemia among children in endemic regions and the diminished iron absorption that accompanies malaria infection. There are more than 200 million cases of uncomplicated malaria among children in sub-Saharan Africa each year alone. Young children in malaria-endemic areas have multiple, recurring malaria infections throughout childhood, likely leading to varying levels of chronic inflammation. The inflammatory response that accompanies malaria and other infections leads to increased production of the hepatic protein hepcidin, which cause the degradation of ferroportin, the iron efflux protein that permits dietary iron to be released from intestinal cells into the circulation and iron that would be recycled from senescent red blood cells to be released from macrophages [68]. Recent work suggests that hepcidin is lowest at the end of a malarial season in areas of seasonal transmission and best predicts the response to iron therapy, causing some to suggest timing the administration of iron to the end of the malaria season [69,70].

Low hepcidin also best predicted successful incorporation of dietary iron into red blood cells in an iron-stable isotope study in Gambian children [71,72]. This study also confirmed that dietary iron incorporation—an indirect measure of absorption—into red blood cells is diminished in children recovering from post-malarial anemia as compared with children recovering from iron-deficiency anemia alone. However, children recovering from malarial anemia had a greater Hgb gain than children recovering from iron-deficiency anemia, leading researchers to conclude that immediate iron needs in these children were initially met by iron trapped in reticuoendothelial stores during the inflammation of malaria, but then released for supporting Hgb synthesis following antimalarial treatment [71]. 

Delaying the start of iron therapy until after effective treatment of malaria and an accompanying reduction in inflammation and hepcidin thus may be another strategy to safely increase iron absorption and utilization. We recently reported that iron therapy begun 28 days after antimalarial treatment in children with iron deficiency and malaria was more than twice as well incorporated (16.5% vs. 7.9%) as iron therapy that was started concurrently with antimalarial treatment per the current WHO standard of care [73]. In accordance with the greater incorporation, hepcidin concentrations were also significantly lower in the delayed iron group as compared with the immediate iron group at day 28. At day 56, after all children had received the same length of iron therapy, Hgb and iron markers (ferritin, soluble transferrin receptor, ZnPP) were equivalent between the two groups. 

An additional finding was that children in the delayed iron group had a significantly lower incidence of all-cause sick-child visits to the study clinic during the 56-day follow-up period [74]. The most common diagnosis was upper respiratory infection, followed by malaria. Although the mechanism behind the lower morbidity associated with delayed iron therapy is unclear, it follows that the greater percentage iron incorporation that we observed with delayed treatment would be accompanied by less unabsorbed iron in the intestinal lumen. In multiple studies of iron-fortified micronutrient powder, unabsorbed iron has been associated with a shift in the composition of the intestinal microbiome of young children living in malaria-endemic areas, shifting from predominant beneficial barrier strains (e.g., bifidobacteriaceae), to more pathogenic strains (e.g., enterobacteria), and leading to intestinal inflammation [75,76,77].

Recent work suggests that this shift to pathogenic strains may be mitigated by the addition of prebiotic galacto-oligosaccharides (GOS) to micronutrient formulations. In a randomized controlled trial in Kenyan infants, infants who received daily supplementation with multiple micronutrient powder fortified with 5 mg of iron and GOS had no increase in pathogenic bacteria and a lower incidence of respiratory infections compared to children who received iron-fortified power without GOS [78]. The increased incidence of respiratory tract infections with iron reported in this study [78], the Soofi study [65], and in our recent work [74] is in line with pre-clinical evidence demonstrating that the gut microbiome is an important modulator of immunity. Of note, associations between the gut microbiome and respiratory infections are described, with pathogenic shifts in the gut microbiome associated with an increased risk of a range of respiratory tract infections, including pneumococcal pneumonia [79].

Many questions remain after the Pemba study on how to optimize the iron status in children living in malaria-endemic areas. Recent in vitro evidence suggests that iron-deficient RBCs resist invasion of the malaria parasite [80,81], but iron deficiency cannot be a malaria-control strategy because of the risk it poses to the developing brain described above. Establishment of a safe and effective management strategy to address both iron deficiency and malaria when they coexist is thus a public health imperative. 

### 6.2. Iron Supplementation of Iron-Sufficient Pediatric Populations

As noted previously, iron deficiency is the most common nutrient disorder worldwide. The estimated prevalence is 2 billion cases, a number that exceeds the rate of iodine deficiency for which universal supplementation policies have been implemented. However, given that the world population now exceeds 7 billion, more than 5 billion people are not iron deficient. The situation is further enhanced in developed countries. For example, in the United States, the prevalence of total body iron deficiency is 15% in toddlers, and 10–16% in women of reproductive age [82], which means that the majority population is iron sufficient. Thus, careful consideration must be given to the balance between potential risks and benefits. Studies clearly indicate that iron supplementation of iron-deficient individuals and populations improves iron status, and in some cases, neurodevelopment (see above). Conversely, little, if any, evidence suggests that iron supplementation of iron-sufficient populations improves hematologic or neurodevelopmental status.

Acute toxicity (poisoning) from iron overdose is well described and is characterized by acute liver failure. Small children are at highest risk through accidental ingestion of maternal iron supplements. Questions remain whether routine iron supplementation in therapeutic or preventative doses is a risk to the health of pregnant women and young children. The concerns revolve around the theoretical health risks of iron and the epidemiological and clinical trials in which iron supplementation was associated with adverse outcomes. 

The main theoretical risks of iron supplementation in general, but particularly of iron-sufficient populations are the generation of reactive oxygen species, alteration of the intestinal microbiome toward a more “pathogenic” profile with or without an increase in diarrheal diseases, and an increased risk of non-gastrointestinal infections [83,84]. In contrast to the large preclinical and clinical research literature on the negative effects of early-life iron deficiency, the literature on the potential negative effects of iron supplementation of iron-sufficient populations is relatively limited. Further investigation of the topic seems imperative given the theoretical risks and the small amount of data in humans.

Typically, iron is protein-bound both in the serum, where it is attached to transferrin and other members of the total iron-binding protein family, and in the tissues to storage and chaperone molecules. Non-protein bound iron (NPBI) can mediate cellular DNA damage under prooxidant conditions. NPBI appears in the serum when the iron-binding capacity (TIBC) is overwhelmed, which can occur with a rapid release of iron during hemolysis or with rapid infusions of iron. Since enteral iron uptake is well regulated by the hepcidin system from a very early age, the chances of NPBI being present with enteral iron supplementation are low. Nevertheless, Brittenham et al. have recently demonstrated that in healthy women iron given at the standard treatment dose on an empty stomach can result in measurable NPBI in the serum, although no evidence of oxidative stress was observed [85]. 

Pediatric populations that would be at risk of NPBI and oxidative stress when given enteral iron include premature infants, because of their immature antioxidant systems and low serum transferrin concentrations and consequently, low TIBC. Studies of premature infants given up to 18 mg of iron/kg of body weight daily have failed to demonstrate increased oxidative stress [86]. Only one clinical trial in full-term infants suggests that enteral iron may be detrimental to a relevant health outcome such as neurodevelopment when given to an iron-sufficient population. In one arm of a trial of formula iron supplementation in Chile, Lozoff et al. gave an iron-fortified formula to iron-sufficient infants at 6 months of age and found poorer neurodevelopmental outcomes at 10 years of age, compared with infants given a low-iron formula [87]. Interestingly, the authors indicated that within this trial, it was only the infants who had higher than normal Hgb concentrations that demonstrated the negative neurodevelopmental effects with the iron-supplemented formula. Since the trial was targeted more toward providing iron-fortified formula to a population with a high rate of iron deficiency, supporting evidence regarding NPBI or oxidative stress markers was not available for this unanticipated finding. Nevertheless, this trial has been cited as evidence that enteral iron supplementation to iron-sufficient individuals may be problematic. Preclinical studies to support the notion have been scarce. Iron-sufficient rat pups given iron in doses between 2.5 mg/kg and 30 mg/kg body weight showed increased risk to memory performance as adults as a function of iron dose [88]. Again, no brain-tissue evidence of global or regional iron overload or oxidative stress was provided, making it difficult to assess the causative link between iron dosing and biologically plausible specific brain pathology.

The concern about enteral iron altering the intestinal microbiome to a more pathogenic state mentioned above in the context of malaria-endemic areas also extends to other areas. Prior to the recent advent of microbiome analyses, concerns of iron supplementation increasing the risk of diarrhea from siderophilic organisms such as *E. coli* and Salmonella had been raised. Yip et al. reported no increase in diarrheal disease in US breastfed infants supplemented with iron [89]. The concern about enteral iron supplementation in iron-sufficient individuals with intact hepcidin regulation of intestinal absorption is that an iron-sufficient individual absorbs less than 20% of enteral iron. The unabsorbed iron progresses downstream in the intestine to the colon and results in a high intraluminal iron concentration that fosters the growth of siderophilic bacteria. Lactobacillus, which is gut-protective, is not siderophilic and thrives in a low-iron environment compared with *E. coli*. A recent study in Africa, while performed in children at risk for iron deficiency, emphasized this shift in intestinal microbiota [90]. Other populations that may be at greater risk from this type of microbiome shift include premature infants with their higher risk of necrotizing enterocolitis. No studies have yet assessed whether unabsorbed enteral iron plays any role in this devastating disease. Similarly, whether the risk of non-diarrheal disease, such as upper respiratory tract infections mentioned above, is present in iron-sufficient populations supplemented with iron remains to be tested.

It is reasonable to postulate that the newborn infant is “set up” to require minimal exposure to enteral iron in the first months after birth when it is most vulnerable to infection and yet maintains iron sufficiency at the tissue level in order to maintain growth and development [91]. The appropriate weight for a gestational age full-term infant with no risk factors for iron deficiency during gestation [92] who undergoes “delayed” cord clamping, is breastfed and grows at the standard velocity on WHO curves has enough iron to meet its requirements until at least 4 months and likely 6 months of age. Thus, there appears to be little need for a large source of dietary iron in these otherwise iron-sufficient babies. Indeed, human milk provides very little iron and that iron is tightly bound by lactoferrin and thus not readily available for the pathophysiological processes described above [91].

## 7. Summary and Conclusions

Early-life iron deficiency is common and can negatively affect the brain development of children. Therefore, approaches aimed at reducing the risk of early-life iron deficiency and brain dysfunction are of public health importance. Ensuring maternal iron sufficiency during pregnancy, delayed clamping or milking of the umbilical cord, and promotion of breastfeeding are the most cost-effective approaches for ensuring that the infant begins postnatal life with sufficient iron stores. While current screening and treatment recommendations may suffice for iron-sufficient populations, biomarker-based early screening and treatment strategies may be necessary for those at risk of early-life iron deficiency. Routine iron supplementation is a cost-effective method of improving iron nutrition of at-risk children, but indiscriminant iron supplementation of children in malaria-endemic regions and iron-sufficient populations should be avoided. 
